# Supplementary material for: Role of MicroRNAs and their Downstream Target Transcription Factors in Zebrafish Thrombopoiesis
Source: Res Sq. 2023 Apr 24:rs.3.rs-2807790. Preprint. [Version 1] doi: 10.21203/rs.3.rs-2807790/v1 (PMC10168436; doi:10.21203/rs.3.rs-2807790/v1)
Supplement: Supplement 1 [file NIHPPrs2807790v1-supplement-1.pdf]

# Supplementary Files

This is a list of supplementary files associated with this preprint. Click to download.

- SupplementaryInfoFile.pdf
- SupplementaryDatasetFile.xlsx
